# Food Cravings and Eating: The Role of Experiential Avoidance

**DOI:** 10.3390/ijerph16071181

**Published:** 2019-04-02

**Authors:** Amy J. Fahrenkamp, Katherine E. Darling, Elizabeth B. Ruzicka, Amy F. Sato

**Affiliations:** 1 Mayo Clinic College of Medicine, Rochester, MN 55905, USA; 2 Department of Psychological Sciences, Kent State University, Kent, OH 44224, USA; kdarlin1@kent.edu (K.E.D.); ebollin1@kent.edu (E.B.R.); asato2@kent.edu (A.F.S.)

**Keywords:** late adolescence, experiential avoidance, food craving, eating behaviors, emotional eating, cognitive restraint, weight, body mass index

## Abstract

Food cravings have been associated with problematic eating behaviors, such as emotional eating. Late adolescence is an important developmental period to examine this association, as late adolescents have greater independence in food choices as well as potentially higher demands during a transitional period of their lives. Mechanisms underlying the association between food cravings and problematic eating remain unclear. This study examined whether experiential avoidance (EA) may be one possible mechanism mediating the association between higher levels of food cravings and problematic eating behaviors. Late adolescents (*n* = 174) completed measures assessing EA, food cravings, and three problematic eating behaviors: emotional eating, cognitive restraint, and uncontrolled eating. Height and weight were measured objectively to calculate body mass index (BMI). Food cravings were positively associated with emotional eating and mediated by EA. EA also significantly mediated the association between greater cognitive restraint and greater food cravings. No significant mediation was detected for food cravings and uncontrolled eating. Future research may consider EA as a treatment target in intervention strategies for late adolescents seeking to decrease emotional or restrained eating behaviors.

## 1. Introduction

Food cravings have been associated with problematic eating behaviors, such as overeating, emotional eating, binge eating, disordered eating, or higher weight status [1,2,3,4]. Late adolescence is a salient developmental period to examine food cravings and eating behaviors, as weight gain and unhealthy eating are problematic among late adolescents [3], late adolescents have more autonomy in managing their food intake, and adolescents exhibit more difficulties in behavioral regulation compared to children and adults [4]. Specific mechanisms by which this association functions have not received much attention in the literature. This line of inquiry is important as it may aid in elucidating the complex association that exists between food cravings and problematic eating behaviors, and inform the development of novel interventions producing greater treatment effects. With this background in mind, the present study sought to examine one possible mechanism—experiential avoidance (EA)—by which the association between food cravings and problematic eating may exist among late adolescents. EA refers to a person’s unwillingness to be present in unpleasant situations paired with responses attempting to change their aversive private experience [5].

A food craving is defined as an intense and persisting desire for a specific type of food. By definition, a craving makes it difficult for individuals to withstand non-consumption of their desired food [6,7]. Having a food craving may not inherently be a negative experience. However, 80–85% of food cravings among college students and adults lead to intake of the desired or similar food [7,8] and higher levels of food cravings are associated with problematic eating behaviors in both clinical and non-clinical samples [9,10,11]. Typically, food cravings are assessed via self-report measures, such as the Food Craving Inventory—II, which assesses frequencies of experienced food cravings [12]. Other applicable measures, such as salivary secretion and changes in heart rate, have been used to physiologically assess cravings, including food and alcohol cravings [13,14]. Regardless of the assessment format used, food cravings have consistently been linked to several problematic eating behaviors, including overeating or binge eating and eating after restricting specific foods [1,15]. For example, in a clinical sample of women with bulimia nervosa, food cravings led to binge eating when women experienced negative moods [11]. Understanding food cravings and eating behaviors in adolescents is particularly relevant, as this age group consumes the highest amount of sugar-sweetened behaviors, which show addictive properties as evidenced by an increase in cravings and withdrawal symptoms in overweight and obese adolescents’ (ages 13–18) after a 3-day cessation [16]. To expand our understanding of food cravings in non-clinical samples, the present study focuses on three problematic eating behaviors in late adolescents: uncontrolled eating, cognitive restraint, and emotional eating. Uncontrolled eating refers to the tendency to overeat due to loss of control in eating. Cognitive restraint refers to the tendency to restrict dietary intake in attempt to control weight. Cognitive restraint is conceptualized as a problematic eating outcome in this study due to its associations with overeating and overweight/obesity, yet cognitive restraint may also have benefits in weight management when used without excess. Emotional eating refers to overeating in response to negative affect. These eating behaviors have been assessed using the Three Factor Eating Questionnaire [17].

These three problematic eating behaviors have been associated with negative health outcomes including increased risk for weight gain, disordered eating, poorer self-esteem, and poorer emotion regulation in children and adults [1,18,19]. In one study, women with and without Binge Eating Disorder (BED), monitored triggers of binge eating episodes. Women who did not meet criteria for BED, but still reported binge eating episodes, experienced higher levels of negative affect, perceived loss of control, and higher levels of food cravings, compared to women without BED and no binge eating episodes [1]. More specific to adolescents (ages 10–17), researchers examining emotional eating adolescents’ neural responses suggested that food images produced stronger neural activations than non-food images [20]. These findings illustrate the rationale to examine mechanisms by which obsessive thoughts or cravings for food associate with for problematic eating late adolescents. Limited research has examined the association between food cravings and these problematic eating behaviors within nonclinical samples or the mechanisms by which these associations exist—two gaps in the literature addressed in the present study.

Further clarification of the mechanisms underlying the association between food cravings and various problematic eating behaviors may inform intervention development and improve maladaptive eating behaviors. For example, food cravings may lead to greater levels of emotional eating (i.e., eating in response to a strong affective experience), in part, through inflexible coping styles and a lack of mindful eating behaviors [21]. For example, impulsivity [22] and reactivity to food cues [9] represent constructs that may serve as mechanisms explaining food cravings and eating behaviors. Impulsivity and reactivity to food cues may lead to problematic eating, for example in the context of an individual who exhibits a hasty reaction to a food craving in order to obtain immediate gratification by eating the desired food item. [4]. EA may be related to this process in that individuals with exhibit higher EA may have greater difficulty tolerating distress/discomfort and may pursue immediate actions to avoid that discomfort. Further, EA may uniquely help explain how people with avoidant tendencies may “give in to” food craving and thus exhibit problematic eating behaviors, regardless of their impulsivity or reactivity to food cue itself. Further understanding the pathway to problematic eating behaviors (i.e., via inflexible coping styles, mindless eating) would offer intriguing and potentially robust therapeutic alternatives for the amelioration of these eating behaviors.

Inflexible coping styles may include experiential avoidance, which represents one mechanism by which the association between food cravings and problematic eating behaviors exists. Greater levels of EA have been associated with negative psychological and physical health outcomes including substance abuse and affective disorders [5,23]. More recent research has linked higher levels of EA with greater disordered eating behaviors [24,25,26]. One study showed that EA mediated the association between higher “anxiety sensitivity” (fearing loss of control) and greater levels of disordered eating [24]. Masuda and colleagues (2010) similarly found that greater psychological inflexibility (higher EA) and greater disordered-eating cognitions (e.g., fear of weight gain, importance of being thin or attractive to be socially accepted) predicted poorer psychological health in college students [26]. This research supports the importance of EA within the context of disordered eating, yet more research is needed to examine whether EA is associated with differential problematic eating behaviors among young adults—a central aim of the present study.

Research clearly demonstrates that EA is relevant to providing a more comprehensive understanding of aversive somatic experiences [27]. As such, EA may be important to examine within the context of aversive somatic experiences (i.e., an uncomfortable feeling) central to the operational definition of a food craving. At least one group of researchers have conceptualized food cravings as a food-specific context that draws upon an individual’s practice of psychological flexibility (i.e., acceptance of negative experiences such as anxiety, physical tension, stress, etc.)—terminology closely intertwined with EA [28]. In fact, EA represents a more specific facet to psychological inflexibility [29]. In the context of understanding mechanisms driving responses to food cravings, EA may help explain why a person consumes the food craved or, alternatively, chooses to tolerate an uncomfortable sensation until the craving subsides. Greater propensity to “give in” to (i.e., demonstrating greater EA) uncomfortable private experiences (i.e., food cravings) may subsequently lead to problematic eating behaviors (i.e., emotional eating, uncontrolled eating, etc.) as a means to ameliorate this unpleasant somatic experience.

The hypothesized directionality of effect, however, may not apply equally to all problematic eating behaviors. More specifically, cognitive (or dietary) restraint is hypothesized to lead to increased food cravings. Dietary control strategies involving hunger suppression—restricting food even when hungry—may be temporarily advantageous for weight loss and dieting, but often results in the undesired effect of greater food cravings and greater caloric intake in times of distress [30,31]. Consequently, individuals reporting greater cognitive (hunger) restraint are likely to exhibit greater eating-related avoidance through emotional or hunger suppression [15]. This avoidance strategy may exacerbate their negative experience [32] and result in a stronger food craving and subsequent food intake. That is, within the context of the food craving-cognitive restraint association, EA may be a mechanism by which cognitive restraint is linked to increased food cravings among late adolescents—a hypothesis to be tested in the present study.

## 2. The Present Study

This study builds upon the literature by exploring mechanisms by which food cravings are linked to increased problematic eating behaviors during late adolescence, which is a developmental period with known risks of unhealthy eating, weight change, and behavioral regulation abilities [4,20]. Greater EA has been associated with eating disorder symptomology [33,34], but less research has examined EA and eating behaviors within non-clinical samples. The present study extends prior research through the examination of EA as a potential mechanism through which the experience of food cravings is associated with problematic eating behaviors (i.e., emotional eating, cognitive restraint, uncontrolled eating) among late adolescents. Specifically, we hypothesized that greater EA would mediate the association between greater food cravings leading to greater levels of both emotional eating (Hypothesis 1) and uncontrolled eating (Hypothesis 2). In addition, we predicted that greater EA would mediate the association between greater levels of cognitive restraint leading to increased food cravings (Hypothesis 3).

## 3. Materials and Methods

### 3.1. Participants

The present study involved research from secondary analyses within two highly similar studies examining predictors of weight change in first year college students. Three published articles from Study 1 have presented data from examinations of food insecurity [35], moderating effects of social support with stress eating and weight gain [36], and the moderating effects of BMI with stress and eating [37]. No articles have been previously published from Study 2. The secondary analyses presented in this study from combined Study 1 and 2 data have not been previously published. Studies 1 and 2 included 204 late adolescents who were first year undergraduates from a northeastern Ohio university. The larger studies (Studies 1 and 2) examined predictors of weight gain in first year college students (*n* = 103 in Study 1; *n* = 101 in Study 2). These studies were conducted during three consecutive academic years, beginning in 2012–2013. Recruitment methods for both studies included fliers posted throughout campus and a posting on a campus website listing available research studies for student participation.

Late adolescents were eligible for the present study if they: (1) were first year college students at the university where the study took place; (2) were 18 to 24 years of age; and (3) were fluent in written/spoken English to understand procedures (e.g., consent process) and materials. Given that the focus of the larger studies was to examine normative patterns of weight change among college students not actively trying to change their weight, late adolescents were excluded from participation if they: (1) endorsed clinically significant symptoms of disordered eating on the Eating Disorder Examination Questionnaire (as indicated by self-reported restrictive eating, excessive exercise, or binging/purging behaviors to control weight; [38]; (2) had a BMI in the “underweight” range as defined by the Centers for Disease Control and Prevention (BMI < 18.5 kg/m^2^); (3) were actively participating in a formal weight loss program (e.g., Weight Watchers); (4) had lost more than five pounds in the last month; or (5) reported current food allergies or medication use that would affect their appetite (only for 2013–2015 data collection). The latter exclusion criteria was specific only to Study 2 as this study assessed late adolescents’ food intake during an eating paradigm and these variables presented a risk for influencing the food intake assessment.

Using these inclusion/exclusion criteria, 25 late adolescents were excluded from study participation: one individual was 28 years old, two late adolescents endorsed clinically significant symptoms of an eating disorder (as indicated on the EDEQ), one late adolescent reported that English was not their primary language, eight late adolescents were in the underweight BMI range (<18.5), one late adolescent reported losing more than five pounds in the past month, six late adolescents reported food allergies (e.g., dairy, nuts), and six late adolescents were taking medications that affect their appetite (e.g., psychotropic medications).

In addition, five late adolescents were excluded from these analyses because they did not complete 70% or more of the items on self-report measures. This resulted in a final study sample of 174 late adolescents in the present analyses. No significant demographic differences (i.e., age, gender, race, baseline BMI) were observed from Welch’s t-test and chi-square analyses between the five excluded late adolescents with missing self-report data and the sample included for present analyses. Of the 174 late adolescents included in the present analyses, there were also no significant differences in demographic characteristics (i.e., age, gender, race, baseline BMI) between late adolescents from Studies 1 and 2. However, differences were found in mean total scores of food craving (FCI-II; *t*(170) = −4.75, *p* < 0.001), experiential avoidance (AAQ-II; *t*(170) = 2.27, *p* < 0.05), and cognitive restraint (TFEQ-R 18v2 CR subscale; *t*(157.40) = −2.99, *p* < 0.01) between Study 1 (*M* = 0.86, *SD* = 0.60; *M* = 5.63, *SD* = 1.23; *M* = 5.61, *SD* = 2.15) and Study 2 (*M* = 1.30, *SD* = 0.60; *M* = 5.20, *SD* = 1.24; *M* = 6.68, *SD* = 2.49) samples, respectively.

Of these 174 late adolescents, 76% were female (*n* = 133) and their average age was 18.21 years (*SD* = 0.68). Mean baseline BMI for the overall sample was 25.00 (*SD* = 5.35), with percentages in the following BMI categories: 59.2% healthy weight (between 8.5–24.9) and 40.8% overweight/obese (25.0 and above). The racial distribution for the present sample included 69.5% White, 15.5% Black, 7.5% More than one race, and 7.5% of late adolescents identified as a race outside of those categories. Descriptive characteristics are further presented in Table 1.

### 3.2. Procedure

Both of the larger studies (i.e., Studies 1 and 2) from which data for the present study were collected were approved by the university’s Institutional Review Board. Nearly identical procedures were employed for these studies. Study 2 differed from Study 1 in that late adolescents completed an additional laboratory task to examine the role of stress in relation to weight status and objective food intake was measured. For the laboratory stressor task, late adolescents were given 10 min prior to the speech task to begin completing questionnaires. Then, they were given 10 min to prepare a 5-min speech in which they will pretend they are interviewing for a job. After 10 min, an independent researcher acting as a “judge” informed participants that their speech would be evaluated. Debriefing was conducted with late adolescents at the conclusion of the session. In addition to the stressor task, participants were randomly assigned to receive a pre-weighed amount of cookies at one of four periods throughout the session to measure differences in snack consumption surrounding stress. After researchers obtained informed consent, late adolescents completed a series of self-report questionnaires assessing domains thought to be related to weight management in young adults. Those domains and measures specific to the current study’s hypotheses tested herein are described above (see Measures section). Trained researchers also obtained objective height and weight measurements using a stadiometer and a digital scale. All late adolescents were compensated with either course credit or cash upon completion of each study session.

### 3.3. Measures

#### 3.3.1. Demographics Questionnaire

Late adolescents completed a questionnaire including items regarding demographics, education, medication history, and health to ensure they met eligibility criteria for the study.

#### 3.3.2. Body Mass Index (BMI)

Objective anthropometric measures of late adolescent height (in centimeters) and weight (in pounds) were obtained in the laboratory setting at each assessment time point. Weight was measured using a BWB-800 digital scale (Tanita, Arlington Heights, IL USA) and height was obtained using a stadiometer (Perspective Enterprises, Portage, MI USA). Late adolescents wore light clothing and no shoes for these assessments. Measurements were taken in triplicate and the average value was then used to calculate baseline BMI (kg/m^2^).

#### 3.3.3. Eating Disorder Examination Questionnaire (EDE-Q)

The EDE-Q [38] is a well-validated 41-item questionnaire typically used to screen individuals for an eating disorder. It was used in this study as an eligibility screening tool to exclude individuals who endorsed clinically significant disordered eating symptoms of purging, restraining, or body shape and weight concerns [38].

#### 3.3.4. Acceptance and Action Questionnaire—II (AAQ-II)

The AAQ-II is a 10-item self-report measure assessing broader psychological flexibility (“It’s OK if I remember something unpleasant,” “I’m afraid of my feelings”), which encompasses EA. This study used 7 items from the 10-item measure, based upon a previous validation article by Bond and colleagues (2011), which recommends the use of the 7-item scale [39]. Responses were indicated on a Likert scale ranging from 1 = “never true” to 7 = “always true.” Higher total scores indicate lower EA and higher levels of psychological flexibility. The AAQ-II demonstrates good reliability and validity in previous research [39] and similarly yielded good internal consistency in the present study (α = 0.90).

#### 3.3.5. Food Craving Inventory—II (FCI-II)

The FCI-II [12] assesses self-reported food cravings, described as an intense desire for a particular food that is difficult to resist. A list of 28 foods are given and the individual rates the experience of craving each food using a 5-point Likert scale ranging from 1 = “Never” to 5 = “Always/Almost every day.” Higher FCI-II total scores indicate a greater presence of food cravings experienced in the past month. Similar to previous literature [12] the total score demonstrated excellent internal consistency reliability in this sample, α = 0.93.

#### 3.3.6. Three Factor Eating Questionnaire-Revised (TFEQ-R18v2)

The TFEQ-R18v2 contains 18 items assessing cognitive and behavioral aspects of eating [17]. This validated self-report measure assesses three subscales including Emotional Eating (6 items; “I start to eat when I feel anxious”), Uncontrolled Eating (9 items; “Sometimes when I start eating, I just can’t seem to stop”) and Cognitive Restraint (3 items; “I deliberately take small helpings to control my weight”). Responses are measured on a four point Likert scale ranging from “definitely true” to “definitely false.” Consistent with previous research [17], in this study good reliability was found for the TFEQ-R18v2 subscales: Emotional Eating α = 0.89, Uncontrolled Eating α = 0.80, and Cognitive Restraint α = 0.81.

## 4. Results

### 4.1. Preliminary Analyses and Descriptive Characteristics

Demographic and descriptive data of the self-report measures examined in the mediation analyses are presented in Table 1. Preliminary analyses were also conducted to examine the correlates among the study variables of interest. In the context of self-report measures used in the mediation analyses, higher reported food cravings were associated with lower AAQ-II scores (indicating greater EA; *r* = −0.20, *p* < 0.05), greater emotional eating (*r* = 0.17, *p* < 0.05), greater cognitive restraint (*r* = 0.23, *p* < 0.01), and greater uncontrolled eating (*r* = 0.29, *p* < 0.001). Lower AAQ-II scores (higher EA) were also associated with greater emotional eating (*r* = −0.28, *p* < 0.001), greater cognitive restraint (*r* = −0.28, *p* < 0.001), and greater uncontrolled eating (*r* = −0.20, *p* < 0.01). Only one BMI correlate was found with higher levels of BMI associated with greater reports of emotional eating, *r* = 0.17, *p* < 0.05. Please see Table 2 for detailed correlation table.

### 4.2. Mediation Analyses

**Figure 1:**
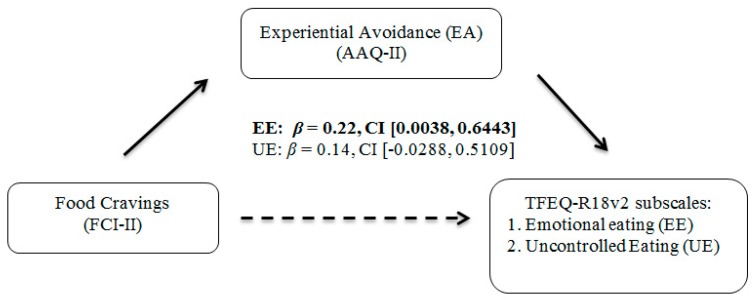
Mediation Analyses Examining EA, food cravings, Emotional Eating, and Uncontrolled Eating among Late Adolescents (Hypothesis 1 and 2). Note. Bootstrapping (with 1000 samples) was used in all mediations. 95% confidence intervals (CI) were employed. If the values between the lower and upper CI do not contain zero, then the mediation is significant. Bolded values indicate significant mediation.

**Figure 2:**
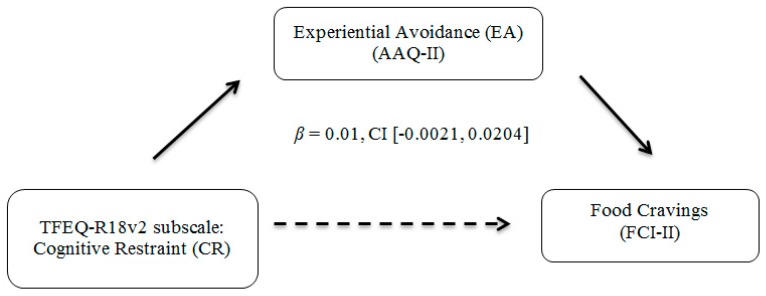
Mediation Analyses Examining EA, food cravings, and Cognitive Restraint among Late Adolescents (Hypothesis 3). *Note.* Bootstrapping (with 1000 samples) was used in all mediations. 95% confidence intervals (CI) were employed. Mediation was not statistically significant.

Mediation analyses were conducted using the SPSS macro PROCESS [40]. Bootstrapping, or drawing repeated samples from the data with replacement to gain multiple estimates of the indirect effect, was used for all mediation analyses. Using this technique bypasses the frequently incorrect assumption of normality for direct effects assumed by Baron and Kenny’s approach [41]. One thousand bootstrapped samples were generated in accordance with previous recommendations [42]. Confidence intervals that do not contain zero indicate significance. Age, race, gender, cohort samples between Study 1 and Study 2, and BMI were controlled for in mediation analyses to examine these mediations above and beyond these potentially confounding descriptive variables. Please see Figure 1 and Figure 2 for the mediation models with corresponding confidence intervals.

First, EA significantly mediated the association between food cravings and emotional eating. There was a significant indirect effect of food cravings on levels of emotional eating through EA, *β* = 0.22, CI [0.0038, 0.6443]. The overall model explained 24.66% (medium effect) of the variance in emotional eating. No significant mediations were found with EA explaining the association between greater food cravings and uncontrolled eating or with EA explaining the association between cognitive restraint and food cravings.

## 5. Discussion

This study sought to examine EA as a potential mechanism that may explain the association between food cravings and problematic eating behaviors during late adolescence, a developmental period characterized by risk for weight gain. As higher levels of EA have been related to poorer health outcomes in previous research, this study hypothesized EA as one mechanism through which food cravings may be associated with problematic eating behaviors. What follows is an overview and discussion of the findings from the present study.

Consistent with hypothesis 1, EA mediated the association between greater food cravings and greater levels of emotional eating. This finding is consistent with previous research showing that adolescents with higher emotional eating self-ratings had stronger neural responses (event-related potentials) when viewing images of food compared to viewing images of other objects [20]. Our study extends this research to suggest that this association (i.e., food cravings-emotional eating) exists in non-clinical populations as well. The effect size was consistent with previous studies examining EA and eating [24,25]. Our results suggest that EA is a potential mechanism for understanding one pathway by which food cravings are linked to emotional eating. Late adolescents who evidence more EA, such as a greater tendency to avoid distress, suppress emotions, or disengage from discomfort, may be more vulnerable to eating food in response to cravings. Put into context, when late adolescents are unable to tolerate the uncomfortable food cravings that may involve intrusive thoughts about specific foods or salivation during cravings, it may be more difficult to allow that craving to subside on its own. Thus, late adolescents may choose to engage in emotional eating to reduce the unpleasant sensation.

Although requiring replication utilizing longitudinal methodology with inclusion of other proposed mechanisms (e.g., impulsivity), the present study’s findings present clinical implications for the development of mechanism-based, therapeutic interventions for problematic eating behaviors. For example, work by Alberts and colleagues found that a seven-week mindfulness-based intervention for adults (ages 28–74)—using acceptance-based instead of control- based strategies—lowered adults’ reported food cravings compared to those not receiving the intervention [43]. Future research may wish to examine the applicability of brief (i.e., mindful eating strategies) or comprehensive (i.e., Mindfulness-Based Eating Awareness Training) acceptance- and/or mindfulness-based interventions for reduction of problematic eating behaviors within obese/overweight or eating disordered populations [44]. Mindfulness-based interventions, such as Mindfulness-Based Stress Reduction for Teens [45], have also been tailored for adolescents. Findings from our study align with an effort to further adapt these interventions to focus on emotional eating targets and complexities in emerging adulthood. It is likely, however, that a different approach may be necessary with respect to the role of EA in explaining the cognitive restraint-food craving association.

Findings from this study were not consistent with the hypotheses that greater EA would mediate the association between food cravings and uncontrolled eating (Hypothesis 2), or that higher levels of EA would mediate the association between cognitive restraint and food cravings (Hypothesis 3). In the context of hypothesis 2, it is notable that binge eating and disinhibited eating—conceptually related to a loss of control in eating—have been associated with food cravings in past research; however, these studies often involved clinical samples of individuals meeting criteria for BED or clinical levels of eating disorder symptoms [12,46]. Findings from this study suggest that the mediating role of EA may be broadly applicable within an emotional eating context but not within the context of uncontrolled eating. Future research is needed to replicate and extend this finding.

In the context of Hypothesis 3, cognitive restraint (or dietary restraint) has been conceptualized as attempts by an individual to restrict their diet for purposes of controlling their weight, leading to hunger suppression, greater food cravings, and subsequent overeating [15,30,31]. One possible explanation for the null cognitive restraint finding in this study may be partially due to the mixed findings regarding the extent that cognitive restraint is beneficial versus problematic in eating- and weight-related outcomes. For example, some research suggests that cognitive restraint is problematic among adolescents (ages 13–15 at baseline) with higher levels of BMI [47]. Further, cognitive/dietary restraint has been associated with negative outcomes including binge eating episodes and weight gain, particularly when paired with negative affect [48]. Higher EA may take the form of thought suppression in cognitive restraint among individuals already having difficulties with eating- and weight-related issues, as thought suppression is a cognitive strategy that is frequently associated with EA [15,30]. Attempts to suppress thoughts about specific foods may in turn lead to food cravings and unhealthy eating. In contrast, this may not be as relevant in evidence-based weight management treatment settings or among non-clinical samples in which cognitive restraint may be helpful in managing weight [48] or does not lead to clinically significant eating issues. Further research is needed to examine the role that EA plays in cognitive restraint and food cravings within clinical samples, as findings from treatment research have shown beneficial reductions in binge eating and weight-related eating issues from implementing acceptance-based and mindfulness strategies [49,50,51]. These treatment studies have primarily involved clinical samples of adults, whereas identifying these issues among late adolescents may help with development and implementation of earlier intervention targets.

The findings of the current study suggest that avoidant tendencies—greater EA—exist within a late adolescent, non-clinical sample and play a role in providing a more comprehensive understanding of unhealthy eating behaviors and the factors maintaining them. However, these findings should be considered in the context of several limitations. First, the mediation results suggested a medium effect size of the overall model. Although, these effects were consistent with previous research examining EA and other eating outcome variables [24,25], future research should consider replication of these findings in adolescents with behavioral eating measures to evaluate reliability of these effects, as well as inferences for clinical and practical significance. Second, although the inclusion of objective measures of height and weight represents a strength of this study, measurements of eating behaviors, EA, and food cravings were based on self-report. Impression management may have influenced late adolescents’ responses related to their eating and levels of food cravings, as they might attempt to portray themselves as healthier. Hunger/appetite may have also impacted their responses on self-report measures, as late adolescents were not given instructions to control for food intake prior to their participation. In addition, the AAQ-II and TFEQ-R18v2 were administered with additional questionnaires in first session of Study 2 (containing the laboratory stress task) to assess constructs outside the scope of this study. Non-eating related questionnaires were administered first, followed by self-reported measures related to eating behaviors, such as the TFEQ-R18v2. Late adolescents’ responses on the self-report measures completed in the session also containing the stressor task, particularly eating-related measures, may have been confounded by demand characteristics from the stressor task. Third, assessing self-reported food cravings illustrates the intensity and types of cravings but does not assess individuals’ behavioral response associated with the cravings (e.g., withstanding or giving into food cravings). Lastly, this study was cross-sectional in design, and future longitudinal or structural equation modeling would be beneficial to formally examine the temporal and causal influences of EA and other related constructs outside of the scope of this study (e.g., impulsivity, food cue reactivity) on the association between food cravings and problematic eating behaviors. The limitations to the use of mediational models utilizing cross-sectional methodology have been well-documented. Although this practice is commonplace within this literature [24,50], the findings described herein are best viewed as the foundational steps for longitudinal research seeking to explicate the associations of food cravings, EA, and problematic eating behaviors and identifying additional mechanisms able to further elucidate these complex associations.

## 6. Conclusions

The present study demonstrates that greater EA may serve as one potentially influential mechanism by which the complex association between food cravings and emotional eating among late adolescents can be better understood. This work may serve as a foundation for future experimental and intervention research. For example, it may be important to incorporate training in acceptance-based strategies (i.e., reducing EA) for late adolescents transitioning to college or gaining more autonomy with their eating to better manage responses to food cravings. Though replication of these findings utilizing longitudinal methodology is necessary, the present study provides the foundational steps to explore how EA may serve as a mechanism by which various somatic symptoms resulting in discomfort (e.g., food cravings, pain) lead to health risks such as weight gain, obesity risk, and other disordered eating pathology for emerging adults.

## Figures and Tables

**Table 1:** Demographic Characteristics and Descriptive Data of Study Sample (*n* = 174). Notes. All percentages may not equate to 100 due to rounding; SD = standard deviation; M = sample mean.

Characteristic	*N* (or *Mean*)	% (or *SD*)	Range
Demographic Characteristics			
Gender			
Female	133	76.4	--
Male	41	23.6	--
Age	*M* = 18.21	*SD* = 0.68	18–23
Race			
White	121	69.5	--
Black or African American	27	15.5	--
Asian or Pacific Islander	4	2.3	--
Native Hawaiian/Pacific Islander	2	1.1	--
American Indian/Alaskan	1	0.6	--
Bi- or multi-racial	13	7.5	--
Other/not identified	6	3.5	--
BMI (baseline)	*M* = 25.00	*SD* = 5.35	18.63–43.54
Self-report Data			Range of mean total scores
Food cravings (FCI-II)	*M* = 1.06	*SD* = 0.64	0–4
Experiential avoidance (AAQ-II)	*M* = 5.44	*SD* = 1.25	1.57–7
Emotional eating (TFEQ 18v2-EE)	*M* = 10.28	*SD* = 3.87	6–24
Cognitive restraint (TFEQ 18v2-CR)	*M* = 6.08	*SD* = 2.38	3–12
Uncontrolled eating (TFEQ 18v2-UE)	*M* = 18.14	*SD* = 4.59	9–31

**Table 2:** Correlation Matrix of Self-report Measures and Demographic Variables in the Present Sample (*n* = 174).

Variable	FCI-II	EA	EE	CR	UR	BMI	Age
Food cravings (FCI-II)	--						
Experiential Avoidance (EA)	−0.20 *	−−					
Emotional Eating (EE)	0.17 *	−0.28 ***	−−				
Cognitive Restraint (CR)	0.23 **	−0.28 ***	0.34 ***	−−			
Uncontrolled eating (UR)	0.29 ***	−0.20 **	0.62 ***	0.25 **	−−		
Baseline BMI (BMI)	−0.09	−0.07	0.17 *	0.09	0.11	−−	
Age	−0.07	−0.04	−0.06	−0.06	−0.07	0.03	−−
Gender	0.03	−0.07	0.28 ***	0.17 *	0.12	0.06	−0.05

Note. *** *p* < 0.001, ** *p* < 0.01, * *p* < 0.05. Bolded values indicate statistically significant correlations.
